# The mental health of elite rugby players (a literature review)

**DOI:** 10.1192/bjo.2021.731

**Published:** 2021-06-18

**Authors:** Kirk Musgrave

**Affiliations:** TEWV NHS Foundation Trust

## Abstract

**Aims:**

Players are Rugby's key asset, what recent research has been conducted into the Mental Health of rugby players/former players?

**Method:**

Initially a Literature Search using HDAS, Ebsco, Researchgate and Googlescholar followed by a Literature Review of relevant articles.

**Result:**

A significantly higher prevalence of anxiety and depressive symptoms in Professional rugby players (compared to the general population) is something that authors agree on. This review considers some of the rugby specific variables not limited to injuries (including concussion), retirement from the sport and finally alcohol abuse.

In 2014, Sullivan looked at the role of potential mediators between concussion and later life depression. Sullivan suggested that the effects of concussion on later life depression may be directly neurological.

Chronic Traumatic Encephalopathy (CTE) is a neurodegeneration which is only definitively diagnosed by post-mortem examination of brain tissue at this time. Today, Chronic Traumatic Encephalopathy is a very controversial subject, for every piece of research which claims to prove CTE, there is another piece of research apparently disproving it.

Alcohol Misuse - Whilst it is well known in general adult psychiatry that alcohol has a significant negative impact on depression and anxiety in the general population, this review summarises findings from research into alcohol misuse in elite rugby players.

**Conclusion:**

In addition to personal variables (which include personality, perfectionism, ability to cope with stress, optimism, pessimism, ability to utilise mental skills, burnout and career satisfaction) there are rugby specific variables which are not limited to injuries, retirement from the sport and finally alcohol abuse.

As mentioned in the paragraph on depression and anxiety, numerous recently published authors agree that a significantly higher prevalence of anxiety and depressive symptoms are seen in Professional rugby players (compared to the general population).

As alcohol misuse has already been researched, there would seem to be an opportunity for future research into the extent of illicit drug use by elite rugby players and potentially the effect of illicit drug use on depressive symptoms and anxiety. As mentioned in the paragraph on depression and anxiety, numerous recently published authors agree that a significantly higher prevalence of anxiety and depressive symptoms are seen in Professional rugby players (compared to the general population).

Finally, given the limited recent published literature on suicide in elite rugby players and former elite rugby players, a significant research gap exists in this particular field.

